# Molecular Dynamics Study on the Diffusion Mass Transfer Behaviour of CO_2_ and Crude Oil in Fluids Produced via CO_2_ Flooding

**DOI:** 10.3390/molecules28247948

**Published:** 2023-12-05

**Authors:** Shuang Wang, Qinglin Cheng, Zhidong Li, Yaming Qi, Yue Liu

**Affiliations:** Key Laboratory of Ministry of Education for Enhancing the Oil and Gas Recovery Ratio, Northeast Petroleum University, Daqing 163318, China; wangshuang517@163.com (S.W.); li1072301502@163.com (Z.L.); qiyaming517@163.com (Y.Q.); 18697068445@163.com (Y.L.)

**Keywords:** CO_2_ flooding-produced liquid, diffusion mass transfer, molecular dynamics simulation, oil and gas gathering and transportation

## Abstract

Carbon dioxide flooding is one of the main methods used to improve crude oil recovery. It can not only improve oil recovery but also reduce greenhouse gas emissions. However, the addition of carbon dioxide makes crude oil become a more complex multiphase fluid; that is, carbon dioxide flooding-produced fluid, in which CO_2_ and various components in crude oil mass transfer each other. This results in significant changes in the structure and properties of crude oil that increase the hazards associated with its gathering and transportation. Therefore, it is very important to explore the microscopic mechanism for the diffusion mass transfer of CO_2_ and crude oil in this fluid, especially during its gathering and transportation. In this study, the diffusion mass transfer process of CO_2_ and crude oil in fluids produced via CO_2_ flooding is studied using molecular dynamics, and the influences of temperature, gas–oil ratio and water content are explored. Observations of the configuration and dynamic behaviour of the system show that after the system reaches equilibrium, the majority of the CO_2_ molecules are distributed at the oil–water interface, and CO_2_ is more prone to diffusing into the oil phase than the water phase. Increases in temperature and water content inhibit, while increases in the gas–oil ratio promote, the diffusion mass transfer of CO_2_ in the crude oil system. The results of this study reveal the mechanism for the diffusion mass transfer of CO_2_ and crude oil in fluids produced via CO_2_ flooding and account for the influence of the water phase, which is consistent with actual production conditions and has certain guiding significance for the safe operation of oil and gas gathering and transportation.

## 1. Introduction

At present, oil field exploitation in China has entered the middle and late stages, and it involves greatly increased difficulty and energy consumption. At the same time, it is facing the pressure of ecological and environmental issues, and a technology is urgently needed to solve this contradiction. Carbon capture, utilisation and storage (CCUS) is one of the key technologies that can be used to cope with global climate change. Additionally, CCUS can be combined with oilfield extraction technology. That is, CCUS-EOR (carbon dioxide capture and storage, and enhanced oil recovery) technology not only improves oil recovery but also makes it possible to bury a large amount of carbon dioxide underground, which plays a role in reducing greenhouse gas emissions. It is the most realistic countermeasure that oilfield enterprises can use to cope with peak carbon and carbon neutralisation [1,2,3].

**Figure 1:**
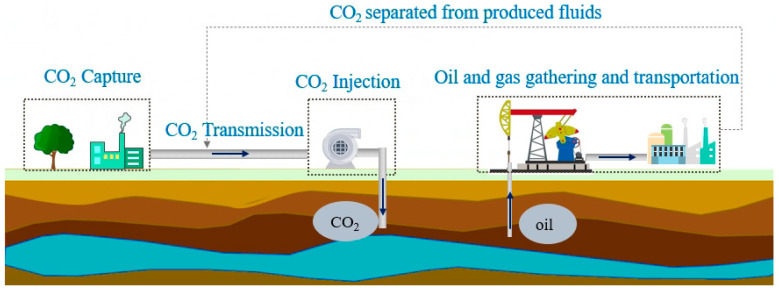
CCUS-EOR technology.

CCUS-EOR technology includes carbon dioxide capture, transportation, injection, sequestration and oil displacement throughout the whole life cycle of oilfield development and greatly promotes the oilfield development process, as shown in Figure 1. After carbon dioxide is injected into the ground, exchanges with the crude oil system occur in the pores of the rock formation, forming a more complex system [4,5,6]. When crude oil with dissolved carbon dioxide is transported through the ground, the changes in pressure and temperature lead to the precipitation of some of the dissolved carbon dioxide, which makes it a more complex multiphase system. In the process of gathering and transportation at the surface, the pressure and temperature are not constant, and the interactions between CO_2_ and each component in crude oil lead to great changes in the structure and properties of the crude oil system. These changes introduce many problems to the current processes for the gathering and transportation of crude oil, such as slug flow and ice blockage, which increase safety hazards and causes harm to the equipment of the gathering and transportation system. Therefore, these issues cannot be ignored for the application of CO_2_ flooding [7,8,9].

To date, many experimental methods have been proposed to study the characteristics of CO_2_–crude oil systems. Hu et al. [10] studied the rheological properties of CO_2_–crude oil emulsions. Through experimental methods, it was determined that the dissolution of CO_2_ reduced the viscosity of the emulsion under low shear and increased the critical shear rate of the viscosity jump. Chen et al. [11] studied the formation and collapse of CO_2_ bubbles in crude oil in pneumatic and decompression experiments. Wu et al. [12] studied the morphology and stability of different CO_2_ emulsions in experiments with slim tubes. A stable CO_2_-in-water (C/W) emulsion formed at 299.15 K, and the emulsion showed the best stability when the water content was 30 vol % and the APG concentration was 3.0 wt %. The structure and morphology of carbon dioxide, oil and water were observed through experimental methods to determine relevant characteristics [13,14,15]. However, the interaction between carbon dioxide, oil and water is microscopic, and the internal mechanism of the change in the morphological properties of the crude oil system caused by carbon dioxide cannot be revealed via macroscopic experiments.

In recent years, molecular simulation has been the main method used to study the microscopic state of matter. Many studies on the characteristics of CO_2_–crude oil systems have been carried out using this method [16,17,18,19]. Wang et al. [20] used molecular dynamics simulation to study the miscibility process of different gases (CO_2_, N_2_, CH_4_ and C_3_H_8_) in nanopores with different crude oils at 413 K and 60 MPa. The effects of gas type, crude oil polarity and chain length on the miscibility process were investigated. Crude oil was more soluble in the CO_2_ phase than it was in the hydrocarbon gas phase and N_2_ phase. Based on the molecular dynamics method, Li B et al. [21] studied the density distribution and adsorption capacity of CO_2_ in systems of resin, resin–asphaltene and asphaltene at different temperatures and pressures, analysed the dissolution mechanism of CO_2_ and revealed the dependence of CO_2_ dissolution on interaction energy. The order of CO_2_ solubility in the three association systems, from high to low, was resin, resin–asphaltene and asphaltene. Li C et al. [22] studied the mutual solubility behaviour of CO_2_ and crude oil during EOR and compared the displacement efficiencies of different gases by calculating the solubility of gas, the volume swelling factor of crude oil, the diffusion coefficient of crude oil and the minimum miscibility pressure (MMP). However, the application of molecular simulations to study the interaction between carbon dioxide and crude oil mostly focuses on reservoir conditions where the temperature and pressure are significantly different from those at the surface where gathering and transportation occur [23,24]. Moreover, there is no relevant research considering the influence of CO_2_ and water content on the diffusion mass transfer of CO_2_ in crude oil. It is necessary to determine the microscopic behaviour of multiphase component diffusion mass transfer in fluids produced via CO_2_ flooding under conditions for the gathering and transportation of crude oil.

Based on the above background, in this study, a molecular model is established to characterise the diffusion mass transfer process of CO_2_ and crude oil in fluids produced via CO_2_ flooding, and molecular dynamics is used to study the diffusion mass transfer process. Thermodynamic and kinetic parameters, such as the RDF, diffusion coefficient and interaction energy, are analysed. The influence of temperature, gas–oil ratio and water content on the diffusion mass transfer is revealed in this paper.

## 2. Results and Discussion

### 2.1. The Effect of Temperature on the Diffusion Process of Carbon Dioxide in the Crude Oil System

The dynamic behaviour of each carbon molecule determines the properties of the produced liquid, thereby affecting the stability and safety of crude oil for gathering and transportation at the surface. Thus, a series of molecular simulations are conducted to study the microscopic dynamic behaviour of each molecule in the CO_2_–crude oil system. Temperature is the key condition that affects the stable and safe gathering and transportation of the produced liquid. To explore the influence of different temperatures on the diffusion process of carbon dioxide in crude oil, according to the actual conditions of oil and gas gathering and transportation, four temperatures are selected for simulation: 283.15 K, 293.15 K, 303.15 K and 313.15 K.

The lower the temperature, the more difficult it is for the system to reach an equilibrium state. If the system reaches equilibrium at the lowest temperature involved in the study, then the system will reach equilibrium at higher temperatures. Therefore, the density of the system and the number of carbon dioxide molecules around the oil (No-c) are calculated at 283.15 K in this study.

**Figure 2:**
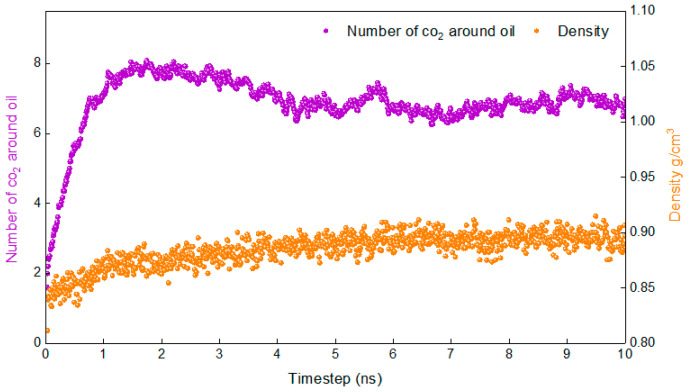
Proof that the simulation is in equilibrium.

Figure 2 shows the variation in the density of the system and the number of carbon dioxide molecules around the oil (No-c) with time at 283.15 K. It is evident that the density fluctuates very little and reaches stability after 1.5 ns, while No-c stabilises only after 5 ns. Based on the above, it can be determined that the system reaches a stable state at 5 ns. It is reasonable to calculate the relevant properties according to the data from 6–10 ns.

#### 2.1.1. Diffusion Process of Carbon Dioxide in Crude Oil

**Figure 3:**
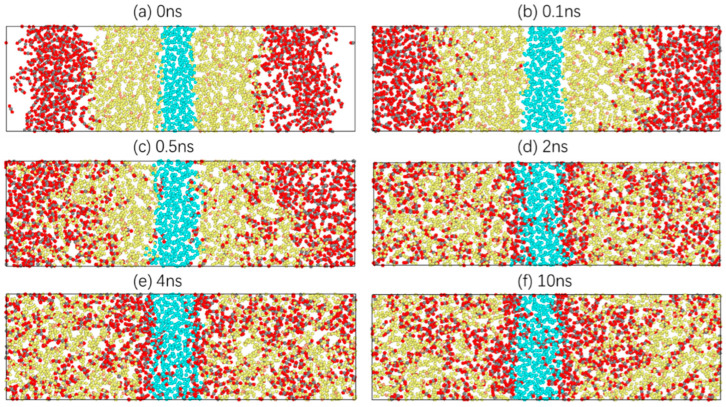
Diffusion process of a CO_2_ molecule in the system.

Taking 303.15 K as an example, the diffusion process of carbon dioxide in crude oil is studied. Information on the atomic velocity, position and model structure is simulated every 10 ps. In total, 1000 model structure images are collected. These images show the dynamic microscopic process of carbon dioxide diffusion in crude oil as the simulation proceeds. In Figure 3, there are six images at moments where there are characteristic changes.

Figure 3a shows the last image of the energy minimisation of the system, which is also the first image of relaxation. CO_2_, oil and water molecules are closely packed, and there is a distinct interface between them. In Figure 3b, CO_2_ molecules gradually diffuse towards the oil phase under a certain pressure, and oil molecules begin to extend towards the gas phase. Subsequently, more CO_2_ molecules enter the oil phase, while a small number of CO_2_ molecules reach the boundary between the oil and water phases and move slightly at the oil–water interface, as shown in Figure 3c. At 2 ns, CO_2_ molecules are dispersed uniformly in the oil phase, with no clear boundary between the CO_2_ phase and the oil phase. At the same time, a CO_2_ molecular layer is clearly formed at the oil–water interface, and a small number of CO_2_ molecules enter the water phase, as shown in Figure 3d. At 4 ns, the system is close to stability, and the CO_2_ molecules in the oil phase tend to aggregate. This is because the distance between the oil molecules increases, resulting in more free space. CO_2_ molecules tend to aggregate under intermolecular forces. Moreover, the thickness of the CO_2_ molecular layer at the oil–water interface increases significantly, and more CO_2_ molecules diffuse into the water phase, as shown in Figure 3e. Finally, Figure 3f shows the stable conformation of the system. The CO_2_ molecules in the oil phase and the water phase move only within a certain range, and the thickness of the CO_2_ molecular layer at the oil–water interface remains basically unchanged.

**Figure 4:**
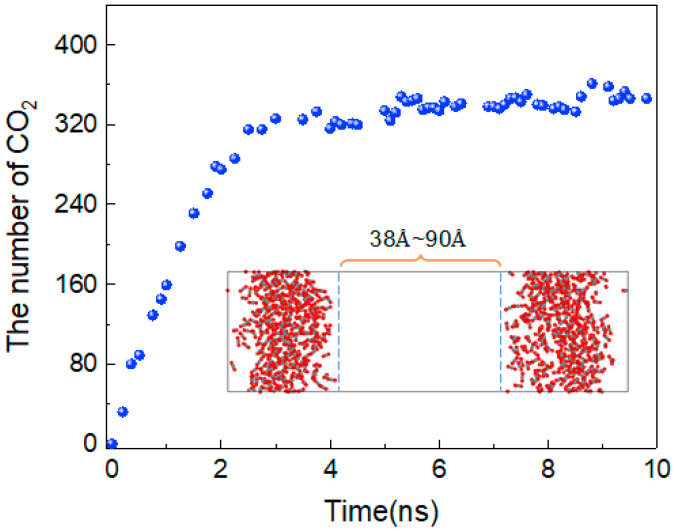
The number of CO_2_ molecules in the oil and water phases.

**Figure 5:**
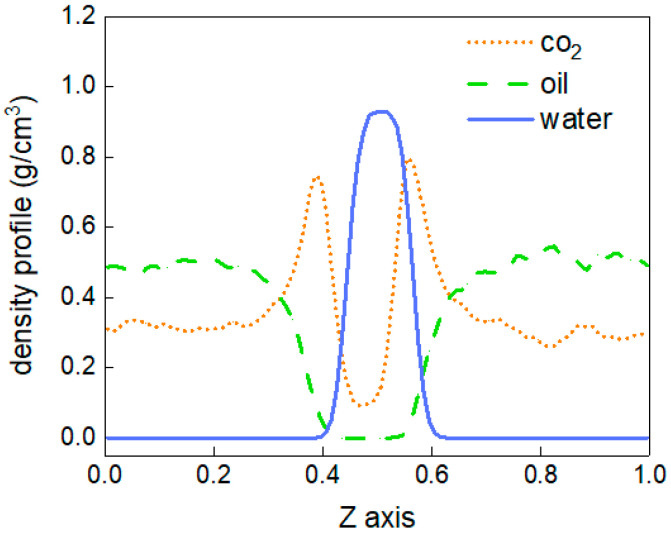
The density distribution at 303.15 K.

To better describe the diffusion process of CO_2_ molecules, the changes in the number of CO_2_ molecules in the oil and water phases are calculated, as shown in Figure 4. The area with a distance of 38 Å~90 Å in the z-direction of the box represents the oil and water phases, and the variation in the number of CO_2_ molecules between 0 and 10 ns in this region is calculated. At 0 ns, there are no carbon dioxide molecules in this region. From 0 to 3 ns, the number of CO_2_ molecules shows linear growth, but after 4 ns, there is little change in the number of CO_2_ molecules, and the diffusion process tends to stabilise. In addition, the density distribution of each molecule in the stabilised system is shown in Figure 5. The orange dotted line represents the density distribution of CO_2_ molecules. Two peaks and valleys appear at the fractional coordinates of 0.4 and 0.6 on the *z*-axis where the CO_2_ molecular density is the highest, which is exactly at the oil–water interface. Second, the values in the ranges of 0~0.35 and 0.65~1.0 are significantly higher than those in the range of 0.4~0.6, indicating that the density of CO_2_ molecules is greater in the oil phase than in the water phase.

The above results show that CO_2_ diffuses into oil more easily, and the force is greater at the oil–water interface than is the force exerted by oil and water molecules on CO_2_ molecules.

#### 2.1.2. The Effect of Temperature on the Diffusion Process

To compare the differences in CO_2_ diffusion in crude oil at different temperatures, the microscopic kinetic properties of the system are described based on the distribution, structure and velocity of molecules. This paper uses the density distribution, radial distribution function and mean square displacement of CO_2_ molecule to characterise CO_2_ diffusion in crude oil.

**Figure 6:**
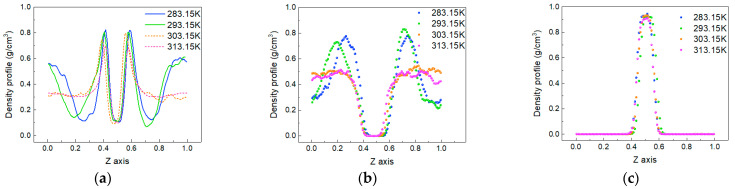
The density distributions of molecules in the system at different temperatures. (**a**) CO_2_, (**b**) oil and (**c**) water.

To explore the change in the distribution of each phase in the system caused by temperature, the density distribution of each phase at each temperature is calculated, as shown in Figure 6. The density distributions of CO_2_ molecules and oil molecules obviously vary with the change in temperature, while there is very little change in the density distribution of water molecules. The results show that the characteristics of the density distribution are mainly reflected at the oil–water interface and the area where the gas phase and oil phase mix.

Figure 6a shows the distribution of the CO_2_ phase. The peak value at the oil–water interface decreases with increasing temperature. This is because the increase in temperature intensifies the thermal motion of molecules, making more CO_2_ molecules enter the oil and water phases. However, the oil and water molecules at the boundary become more distant due to the intensification of thermal motion, reducing the mutual force and weakening the attraction to CO_2_ molecules. In the area where the CO_2_ phase and oil phase mix, there is an inconsistent trend with the change in temperature.

At 283.15 K and 293.15 K, the density of CO_2_ molecules undergoes significant changes, and there is a trough in the density distribution. However, at temperatures of 303.15 K and 313.15 K, the density distribution of CO_2_ molecules becomes relatively flat. In Figure 6b, the oil molecule appears as a peak at the same position at 283.15 K and 293.15 K, and at 303.15 K and 313.15 K, similarly to CO_2_ molecules, the density distribution is flat. This means that at low temperatures (283.15 K and 293.15 K), some oil molecules gather together at a certain location, and CO_2_ molecules are squeezed into other remaining spaces.

**Figure 7:**
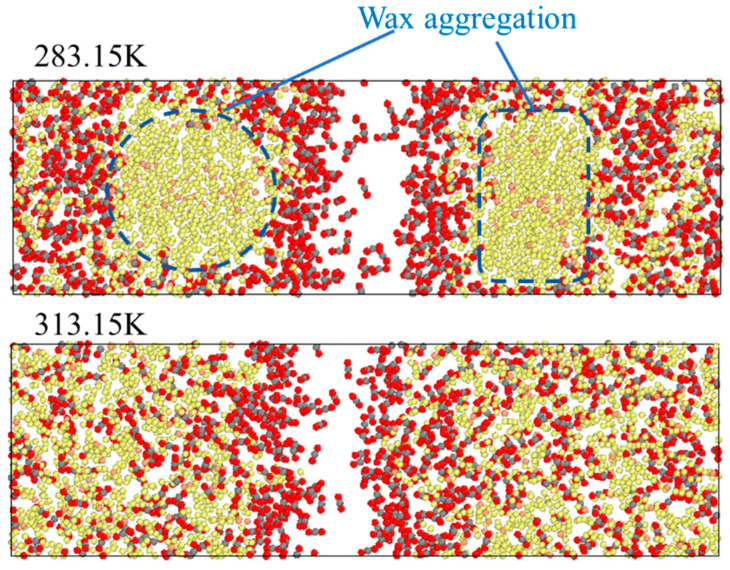
Images of the stable system at 283.15 K and 313.15 K.

To further analyse the reasons for this behaviour, a comparison is made between the images when the system is stable at 283.15 K and 313.15 K. As shown in Figure 7, at 283.15 K, some long-chain oil molecules are closely arranged and complex, forming large clusters through cross-linking and aggregation, with almost no free space, which makes it difficult for CO_2_ molecules to move in the areas of the clusters. This also indicates that the wax precipitation point of oil is in the range of 293.15 K~303.15 K [25].

**Figure 8:**
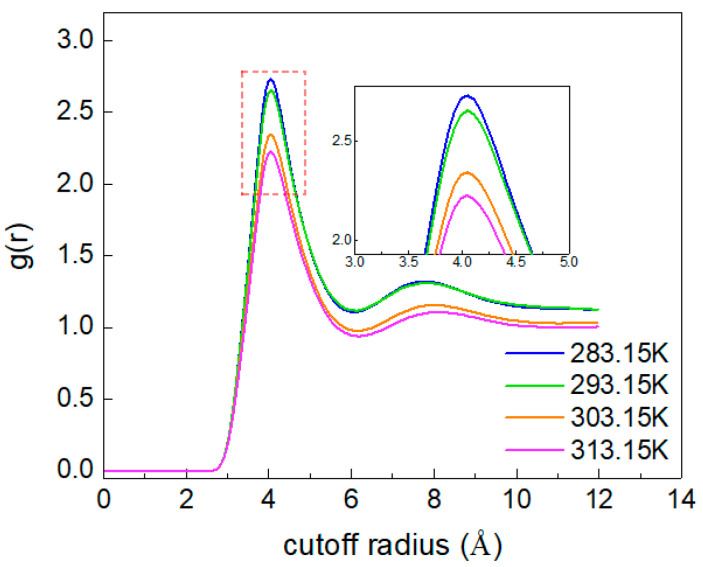
The RDF of CO_2_(C)-CO_2_(C) at different temperatures.

The RDF (radial distribution function) describes the probability of a particle appearing around the central particle. To describe the overall structure and aggregation degree of CO_2_ molecules, the RDF of CO_2_ (C)-CO_2_ (C) is calculated. As shown in Figure 8, with increasing temperature, the position corresponding to the g(r)_C-C_ peak at 4 Å does not change, while the peak value of g(r)_C-C_ decreases. This indicates that the increase in temperature enhances the irregular movement of CO_2_ molecules, making the distance between molecules larger, and loosens the overall distribution structure. Additionally, when the temperature is 293.15 K, the extent of the increase in the g(r)_C-C_ peak value suddenly increases, which indicates that the temperature causes changes in the structure of different phases in the system that result in the aggregation of CO_2_ molecules, which is consistent with the analysis results shown in Figure 6.

**Figure 9:**
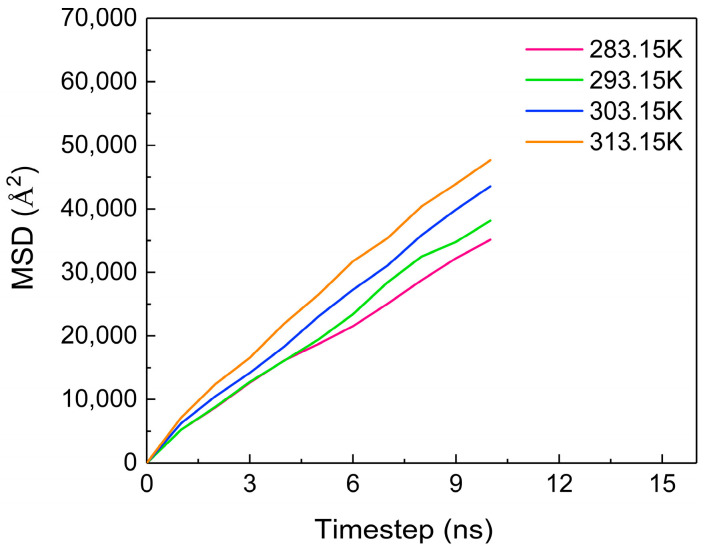
The MSD of CO_2_ at different temperatures.

The diffusion coefficient is used to represent the diffusion ability of the material, which is 1/6 of the slope of the curve of the mean square displacement vs. time [26,27]. Figure 9 shows the MSD (mean square displacement) of CO_2_ molecules at different temperatures, revealing an increasing trend as the temperature rises. The diffusion coefficients of CO_2_ molecules at each temperature are obtained via calculations, as shown in Table 1. As the temperature increases, the diffusion coefficient of CO_2_ molecules gradually increases, which is directly proportional to the temperature change. This is because at higher temperatures, molecules exhibit more vigorous irregular movement, resulting in an enhanced diffusion capability.

Based on the above results, it can be concluded that the increase in temperature can aggravate the thermal motion of CO_2_ molecules, thus increasing the distance between CO_2_ molecules, and oil and water molecules, reducing the van der Waals force between molecules, and inhibiting the diffusion of CO_2_ molecules in the oil phase.

### 2.2. The Effect of the Gas–Oil Ratio on the Diffusion Process of Carbon Dioxide in the Crude Oil System

**Figure 10:**
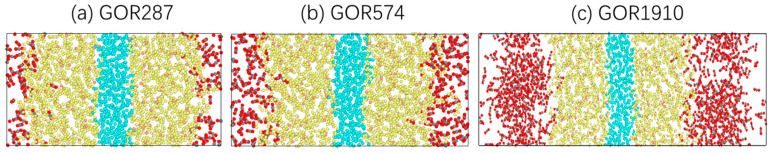
Three gas–oil ratio models.

In actual production, the gas–oil ratio of the produced liquid varies significantly in the early and late stages of development, ranging from tens to hundreds. Whether this significant change has an impact on the diffusion process of carbon dioxide in the crude oil system is a thought-provoking question. To solve this problem, a series of molecular simulations are conducted to explore the effect of the gas–oil ratio on the diffusion of carbon dioxide in the crude oil system. The gas–oil ratio of this block is mainly distributed in the range of 96~1910 SCF/STB. Therefore, 287 SCF/STB, 574 SCF/STB and 1910 SCF/STB are selected for comparison. Figure 10 shows the system models for three different gas–oil ratios.

**Figure 11:**
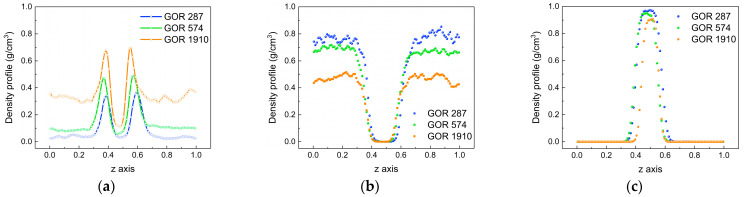
Density distributions of molecules for different gas–oil ratios. (**a**) CO_2_, (**b**) oil and (**c**) water.

To compare the diffusion process for different gas–oil ratios, the density distribution of each phase is calculated using the same simulation method at 313 K. Figure 11 illustrates the density distributions of CO_2_ molecules, oil molecules and water molecules, showing significant variations with the temperature change. This indicates that different gas–oil ratios can indeed influence the diffusion process. As shown in Figure 11a, the density distributions of CO_2_ molecules for different gas–oil ratios are displayed. With an increase in the gas–oil ratio, the peak value at the oil–water interface significantly increases, indicating that the increase in the proportion of CO_2_ molecules promotes their movement. At the same time, the density distribution difference of CO_2_ molecules in the oil phase and water phase gradually increases, revealing that the diffusion difference of CO_2_ molecules in the oil phase and water phase is more obvious at a larger gas–oil ratio. Figure 11b,c show the density distributions of oil molecules and water molecules for different gas–oil ratios. The density of oil molecules and water molecules decreases with an increase in the gas–oil ratio. This is because the higher concentration of CO_2_ intensifies molecular thermal motion and causes it to aggregate at the oil–water interface. It also makes CO_2_ molecules more easily enter the oil and water phases, resulting in larger spacing between oil molecules and a decrease in the density of the oil phase as well as in water molecules.

**Figure 12:**
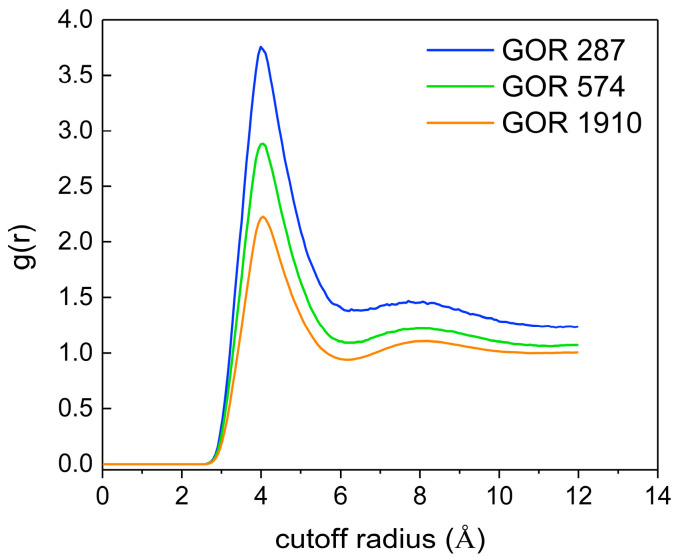
The RDF of CO_2_ (C)-CO_2_ (C) at different gas–oil ratios.

To further explore the distribution of CO_2_ molecules, the RDF of CO_2_ (C)-CO_2_ (C) at different gas–oil ratios is calculated. As shown in Figure 12, with an increase in the gas–oil ratio, the peak value of g(r)_C-C_ decreases, indicating that a higher gas–oil ratio promotes the irregular motion of CO_2_ molecules, which causes an increase in the intermolecular distance and a looser overall distribution structure.

**Figure 13:**
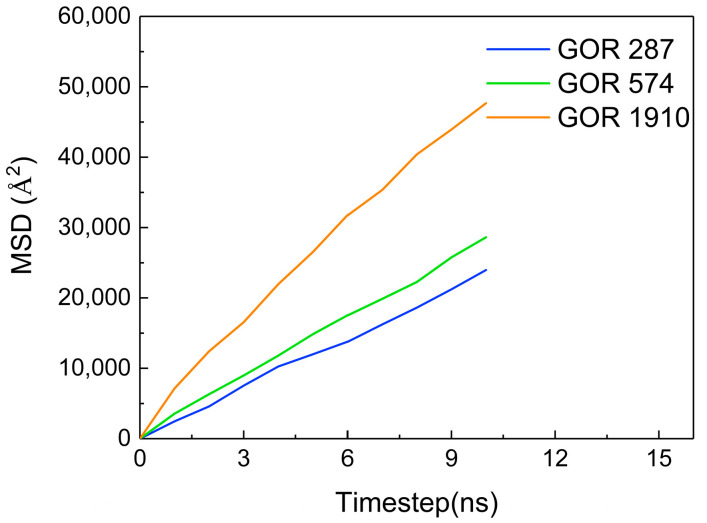
The MSD of CO_2_ at different gas–oil ratios.

Additionally, the MSD of CO_2_ molecules is calculated at different gas–oil ratios, as shown in Figure 13. The MSD gradually increases with an increase in the gas–oil ratio. The diffusion coefficient of CO_2_ molecules in each gas–oil ratio system is determined Using the slope of the MSD curve, as shown in Table 2. As the gas–oil ratio increases, the diffusion coefficient of CO_2_ molecules gradually increases, which is directly proportional to the change in the gas–oil ratio. This is because the higher the gas–oil ratio is, the stronger the driving force is, thereby enhancing the diffusion capacity of CO_2_ molecules.

**Figure 14:**
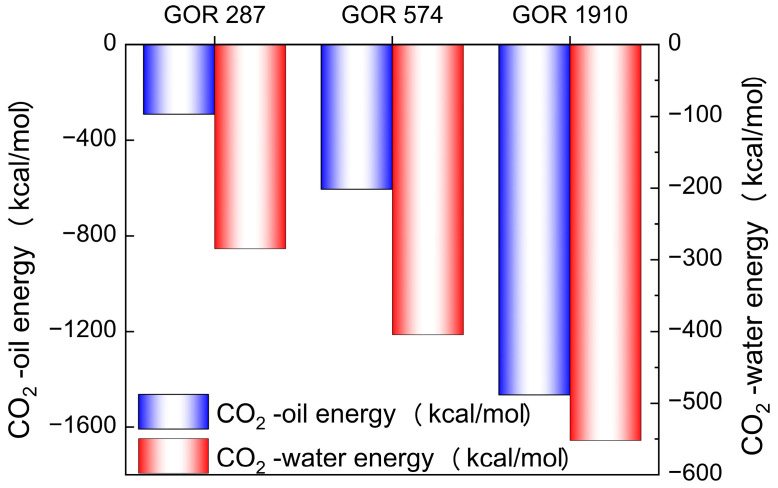
The interaction energy of CO_2_ with oil and water in systems with different gas–oil ratios.

To explain the intrinsic mechanisms of different diffusion processes, the interaction energies between CO_2_ and oil, as well as water, are calculated. E_CO2-oil_ and E_CO2-wate_ represent the interaction energies between CO_2_ and oil, and CO_2_ and water, respectively. Figure 14 shows the E_CO2-oil_ and E_CO2-wate_ in systems with different gas–oil ratios, where a negative value represents intermolecular attraction. The E_CO2-oil_ is much greater than the E_CO2-oil_, which explains why CO_2_ molecules are more prone to diffusing into the oil phase. At the same time, the E_CO2-oil_ and E_CO2-wate_ gradually increase with an increasing gas–oil ratio. This is because a higher gas–oil ratio promotes the movement of CO_2_ molecules, reducing the distance between CO_2_ molecules, and oil and water molecules, thereby enhancing their mutual interaction.

Through the above analysis, it can be concluded that the increase in the gas–oil ratio can enhance the E_CO2-oil_ and E_CO2-wate_, thus promoting the diffusion of CO_2_ molecules in the crude oil system.

### 2.3. The Effect of Water Content on the Diffusion Process of Carbon Dioxide in the Crude Oil System

In actual production, carbon dioxide and water flooding are often alternately performed, which can effectively prevent gas channelling and improve oil recovery. However, it can significantly increase the water content of the produced liquid and have a negative impact on gathering and transportation processes. With the change in water content, the combination of oil and water differs greatly. When the water content increases to more than 60%, the phase inversion characteristics of the produced liquid are affected, causing it to transform from water-in-oil (W/O) to oil-in-water (O/W), thus affecting the diffusion process of carbon dioxide in the crude oil system. The water content involved in the above study is 20%. At this water content, the type of emulsion is water-in-oil (W/O). To further explore the influence of water content on the CO_2_ diffusion process, a water content of 70% is selected as the research condition. At this time, the emulsion system undergoes a phase transition and changes to an oil-in-water emulsion, which can be suitably compared with the previous working conditions.

**Figure 15:**
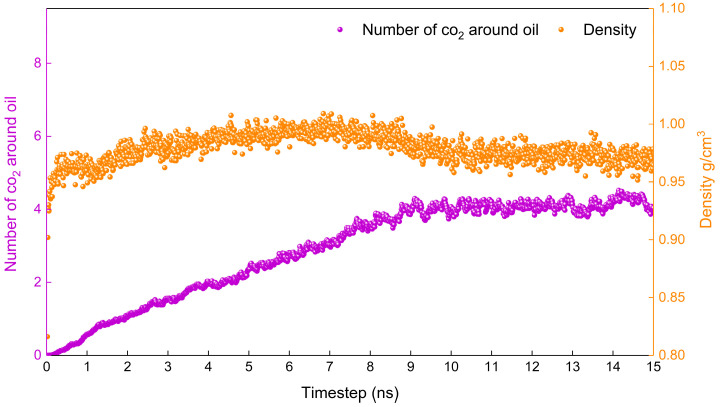
Verification of simulation equilibrium.

Due to the large number of molecules in the oil-in-water system, the system may not necessarily reach the equilibrium state when the simulation time is 10 ns, and thus equilibrium verification is also needed. Figure 15 shows the changes in the density of the system and the number of carbon dioxide molecules (No-c) around the oil at 283.15 K. It is evident that density and No-c still fluctuate significantly before 10 ns, and that the system at this point has not reached equilibrium. Starting at 10 ns in the simulation, the two parameters become stable. Therefore, it is necessary to collect the data after 10 ns when calculating the related properties. In this paper, the equilibrium data at 10–15 ns are used for calculation.

**Figure 16:**
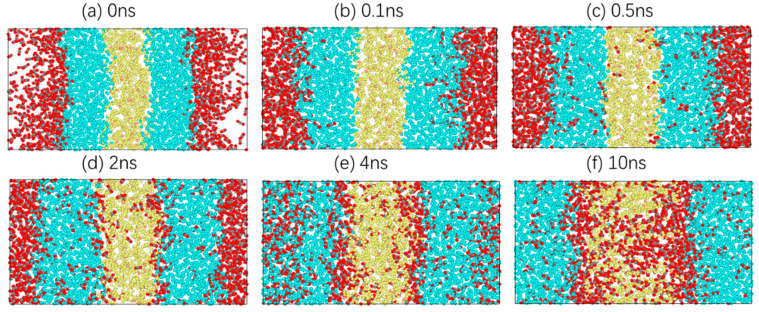
The diffusion process of CO_2_ molecules in the O/W system.

Similarly, the microscopic behaviour of each molecule in the O/W system is studied, and six images at moments with characteristic changes are selected, as shown in Figure 16.

The diffusion process of CO_2_ molecules in the O/W system is different from that of CO_2_ molecules in the W/O system. CO_2_ molecules gradually diffuse into the water phase, but the expansion amplitude of the water phase is relatively small, as shown in Figure 16b. As an increasing number of molecules diffuse into the water phase, CO_2_ molecules disperse homogeneously in the water phase. However, there is still a clear interface between the CO_2_ phase and the water phase where a very thin layer of CO_2_ molecules is formed. More CO_2_ molecules enter the oil phase. The volume of the oil phase begins to expand, as shown in Figure 16c,d. Subsequently, most of the CO_2_ molecules disperse homogeneously at the oil–water interface. The thickness of the CO_2_ molecular layer at the oil–water interface increases significantly, and the number of CO_2_ molecules in the oil phase increases, as shown in Figure 16e. Finally, CO_2_ molecules in the water phase continue to diffuse to the oil phase, and only a small number of CO_2_ molecules are in the water phase. Figure 16f shows the stable conformation of the system.

**Figure 17:**
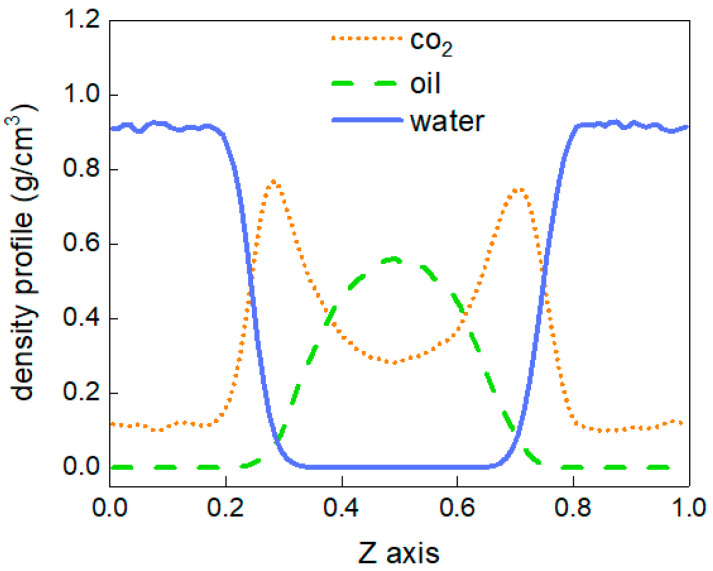
Density distribution of the O/W system.

The density distribution of each molecule in the system after stabilisation is shown in Figure 17. The orange dotted line represents the density distribution of CO_2_ molecules. There are two peaks and valleys at the fractional coordinates of 0.3 and 0.7 on the *z*-axis, that is, the oil–water interface, where the CO_2_ molecular density is the largest. Second, the values in the range of 0.3~0.7 on the *z*-axis are significantly larger than those in the range of 0~0.3 and 0.7~1, indicating that the density of CO_2_ molecules is higher in the oil phase than the water phase. This is consistent with the results of the O/W system, but the water phase has a certain hindrance effect on the diffusion of CO_2_ molecules in the oil phase and on the expansion of the oil phase.

Based on the above analysis, water content has a significant impact on CO_2_ diffusion. To further analyse the influence of water content, the diffusion process of CO_2_ molecules in the system is compared at water contents of 0%, 20% and 70%.

**Figure 18:**
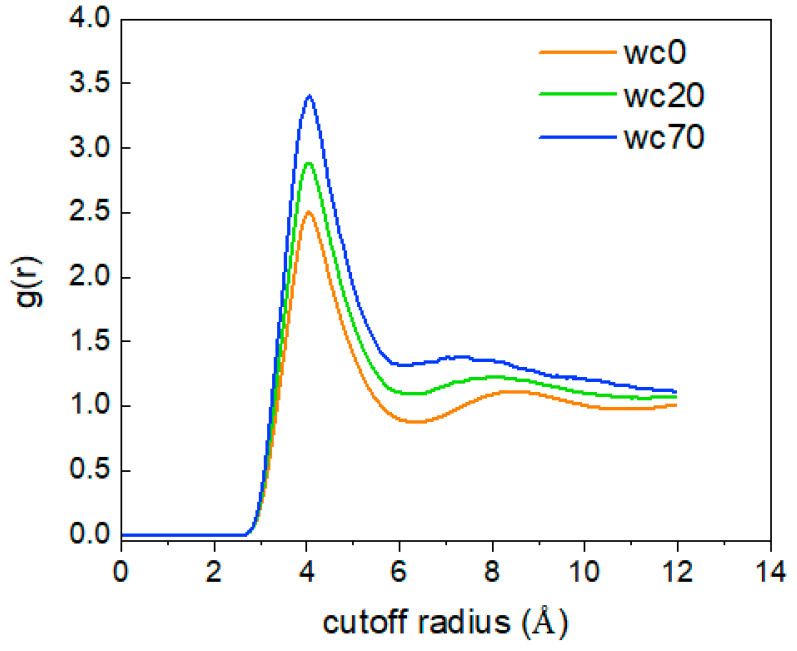
The RDF_CO2(C)-CO2(C)_ of at different water contents.

Figure 18 shows the g(r)_CO2(C-C)_ in the O/W systems at different water contents. As the water content increases, the peak value of g(r)_CO2(C-C)_ becomes larger, indicating that the increase in water content lessens the random motion of CO_2_ molecules, leading to a decrease in intermolecular distance and a tighter overall distribution structure.

**Figure 19:**
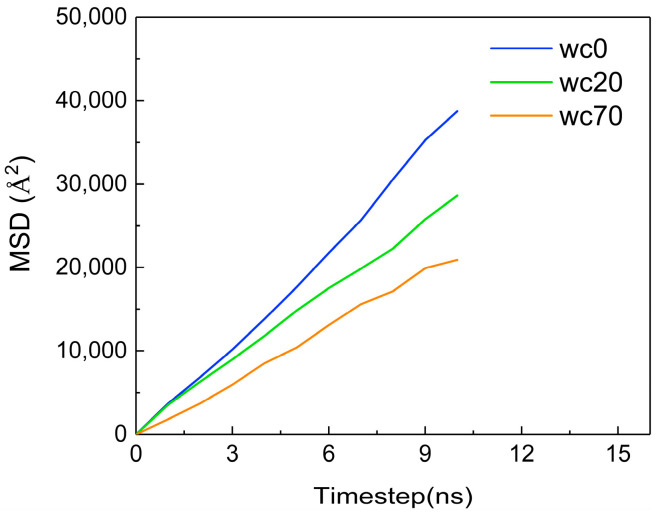
The MSD of CO_2_ at different water contents.

Figure 19 shows the MSD of CO_2_ molecules at different water contents. The MSD decreases with increasing water content. The diffusion coefficients of CO_2_ molecules for each water content can be calculated, as shown in Table 3. As the water content increases, the diffusion coefficient of CO_2_ molecules gradually decreases, which is inversely proportional to the change in water content. This indicates that the outer distribution of the water phase decreases the diffusion of CO_2_ molecules. The reason is that water molecules are tightly arranged and have strong intermolecular interactions. The interaction between CO_2_ molecules and water molecules is weaker than that between water molecules, which decreases the diffusion of CO_2_ molecules in the water phase. At the same time, the water phase limits the diffusion of the oil phase, and the reduction in free space decreases the diffusion of CO_2_ molecules in the oil phase.

**Figure 20:**
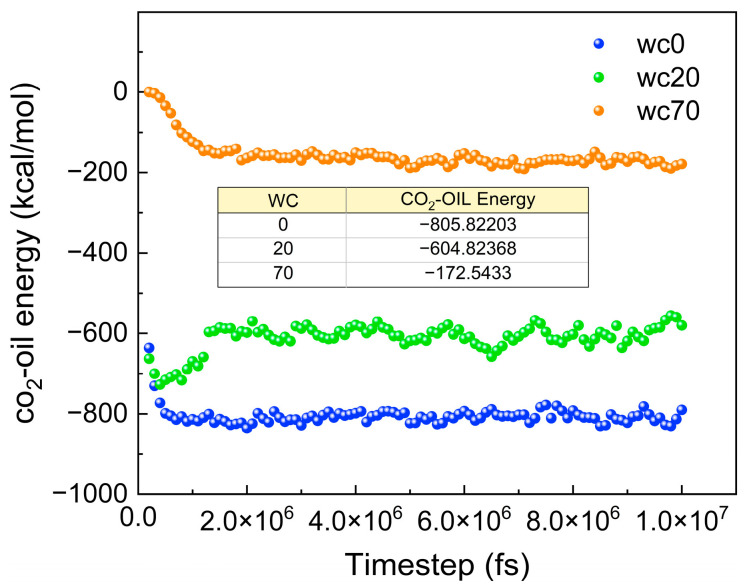
The E_CO2-oil_ for different water contents.

The basis for molecular motion is intermolecular interactions. Figure 20 shows the E_CO2-oil_ for different water contents. The E_CO2-oil_ decreases with increasing water content, which further proves that an increase in the water content decreases the diffusion of CO_2_ molecules.

## 3. Model

**Figure 21:**
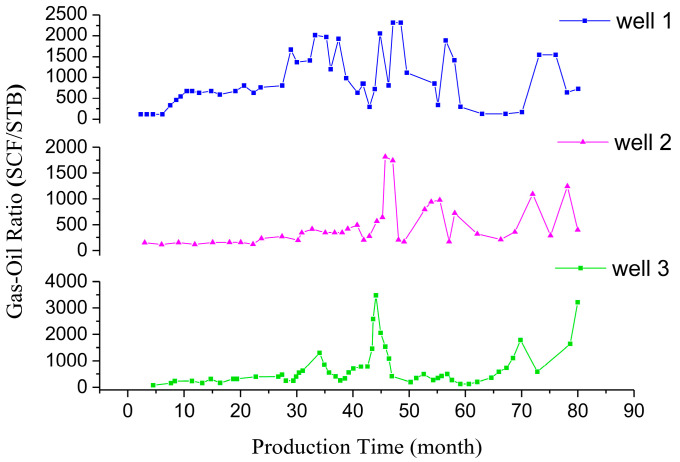
Gas–oil ratio of the extracts.

**Figure 22:**
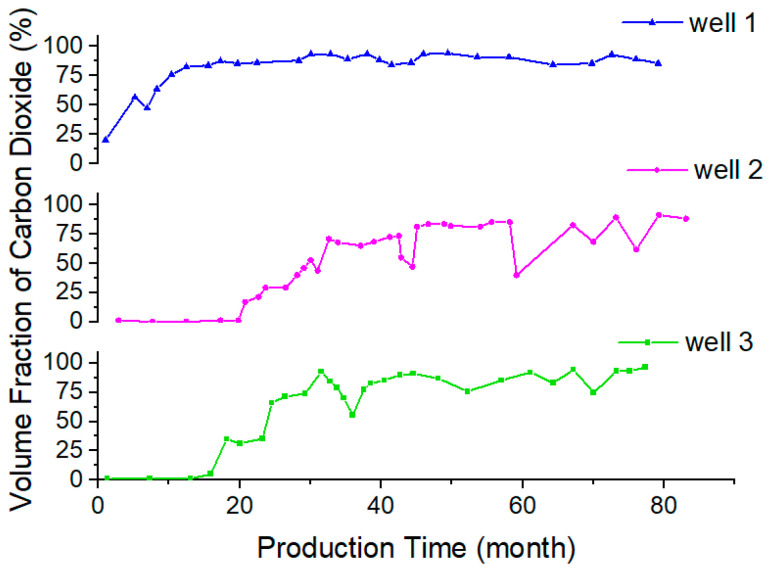
The change in carbon dioxide content in produced gas.

With the use of carbon dioxide flooding, the gas content of oilfield-produced fluid is increasing. The content and composition of gas in crude oil were determined via an oil field test. Figure 21 shows the gas–oil ratio of three production wells in a block with the change in production time. The gas–oil ratio is mainly in the range of 96~1910 SCF/STB and the highest value reaches 3820 SCF/STB. The volume content of CO_2_ in the gas of the produced liquid is usually more than 80%, and can be as much as approximately 98%, as shown in Figure 22. The density of crude oil also shows a trend of first decreasing and then increasing during the development process, which is caused by the extraction properties of carbon dioxide. The pH value of the produced fluid decreases, generally, from 9 to approximately 8. In general, liquids produced via the injection of carbon dioxide have the characteristics of high gas–liquid ratio, foaming and acidity [28,29,30].

**Figure 23:**
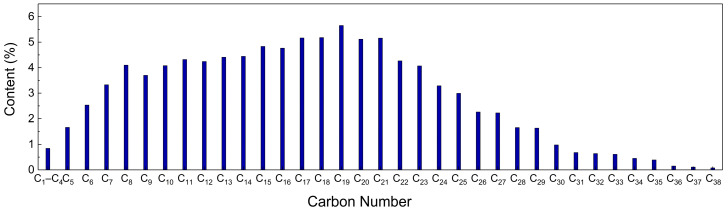
Results of the chromatographic analysis of oil.

**Figure 24:**
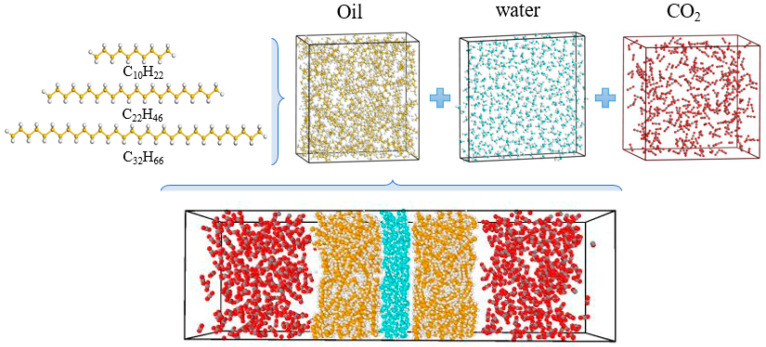
Carbon dioxide–oil–water multivariate model.

The fluid produced via flooding with carbon dioxide mainly includes two components: crude oil and carbon dioxide; water is considered to be in the crude oil. Therefore, the model of fluid produced via carbon dioxide flooding consists of three main components: crude oil, carbon dioxide and water. First, three component models are established and then combined into a carbon dioxide–oil–water multivariate model to characterise the fluid produced via carbon dioxide flooding. The oil composition model is constructed based on the chromatographic analysis results of the well 1 oil sample. The results show high contents of light oil (<C_17_) and short-chain wax (C_17_–C_30_), as shown in Figure 23. To simplify the model, C_10_ is selected to characterise light oil molecules, C_22_ and C_32_ are selected to characterise short-chain and long-chain wax molecules, respectively, and the number of molecules of each component is determined based on the proportion of each component in the results of the chromatographic analysis [31,32]. For the water component, the TIP4P/2005 model is used, which introduces a virtual charged atom on the basis of the original three-site model. During the simulation, the Fix shake command is used to maintain the rigidity of the bond lengths and bond angles [33]. For the CO_2_ component model, the EPM2-flex model is adopted, which is a three-site model considering the flexible deformation of carbon dioxide molecules. This model can accurately describe the structure and properties of carbon dioxide molecules [34,35,36]. The above component models are combined to construct a gas–liquid multiphase model. The oil and water layers are distributed in the middle of the box, and CO_2_ molecules are on both sides of the box with the dimensions 40 × 40 × 133 Å^3^. Each molecule and the multivariate model are shown in Figure 24.

## 4. Simulation Methods and Details

### 4.1. Force Field

The molecular force field is a mathematical model describing the intermolecular force, and it is one of the most important components of molecular simulation. According to the selected force field, the velocity, displacement and other related parameters of molecules can be calculated to obtain the structure, dynamic behaviour and thermodynamic properties of the whole system [37,38]. The total molecular potential energy includes nonbonding potential energy and bonding potential energy. The nonbonding potential energy includes the van der Waals force and Coulomb potential. Van der Waals forces generally comprise Lennard Jones (LJ) potential energy [39,40]. The bonding potential energy includes the bond expansion term, bond bending term and bond torsion term, as shown in Formula (1):

(1) $$\begin{array}{l} U=U(r)+U(el)+U(b)+U(\theta )+U(\phi )\\ (\begin{array}{l} U(r)=4\varepsilon \left[{\left(\frac{\sigma }{r}\right)}^{12}-{\left(\frac{\sigma }{r}\right)}^{6}\right]\\ U_{el}=\sum_{i,j}\frac{q_{i}q_{j}e^{2}}{4\pi \varepsilon_{0}r_{ij}}\\ U_{b}=\frac{1}{2}\sum_{i}k_{b}{\left(l_{i}-l_{i}^{0}\right)}^{2}\\ U_{\theta }=\frac{1}{2}\sum_{i}k_{\theta }{\left(\theta_{i}-\theta_{i}^{0}\right)}^{2}\\ U_{\phi }=\frac{1}{2}\sum_{i}\left[V_{1}\left(1+\cos\phi \right)+V_{2}\left(1-\cos2\phi \right)+V_{3}\left(1+\cos3\phi \right)+V_{4}\left(1-\cos4\phi \right)\right] \end{array} \end{array}$$

In the equation, σ and ε are the LJ potential energy parameters, σ represents the equilibrium distance between atoms, ε represents the depth of the potential energy curve, r represents the distance between atomic pairs, $k_{b}$ represents the elastic constant of bond stretching and $k_{\theta }$ is the elastic constant of bond angle bending. The LJ interaction constant between two different atoms is calculated according to the mixing law.

(2) $$\sigma_{AB}=\frac{1}{2}\left(\sigma_{A}+\sigma_{B}\right)$$

(3) $$\varepsilon_{AB}=\sqrt{\varepsilon_{A}\varepsilon_{B}}$$

The OPLS-AA force field is used for alkanes. The force field parameters of each model are listed in Table 4.

### 4.2. Simulation Details

First, the energy of the system is minimised to obtain a stable configuration, and then a dynamic simulation of 10 ns is performed under the NPT (isothermal–isobaric) ensemble. The data in the range of 6–10 ns are selected to calculate the structural distribution and thermodynamic properties of the system. Periodic boundary conditions are used in all directions of the box. The Nose–Hoover method is used to control the temperature and pressure in the simulation process. The particle–particle particle–mesh (PPPM) method is used for long-range electrostatic interactions. The cut-off radius is set to 12 Å, and the timestep is set to 1 fs. The simulated pressure is set to 2 MPa, which is a commonly used operating pressure value during the oil and gas gathering and transportation process.

Before calculating the results, it is necessary to confirm whether the simulation reached the equilibrium state. Density, a simple and fundamental indicator for measuring whether a simulation has reached equilibrium, is a necessary rather than a sufficient condition for the system to achieve equilibrium. For a complex system, it is not sufficient to determine whether the system has reached an equilibrium state based on this indicator. It is necessary to introduce an indicator that is more sensitive to the diffusion process. During the diffusion process, there is a significant change in the number of molecules of carbon dioxide around oil, which is necessary as the second indicator to measure whether the system has reached equilibrium.

## 5. Conclusions

In this paper, the microscopic mechanism for the diffusion mass transfer of CO_2_ and crude oil in fluids produced via CO_2_ flooding is studied under conditions for gathering and transportation. The molecular dynamics method is used to study the diffusion mass transfer process under different temperatures, gas–oil ratios and water contents. The following conclusions are obtained:

(1) For fluids produced via CO_2_ flooding, when the mass transfer of CO_2_ reaches stability, most of the CO_2_ molecules are distributed at the oil–water interface, indicating that the force of the oil–water interface on CO_2_ molecules is greater than that of oil and water molecules on CO_2_ molecules. Carbon dioxide molecules are more likely to diffuse into the oil phase than the water phase, indicating that the force of oil molecules on CO_2_ molecules is greater than the force of water molecules on CO_2_ molecules.
(2) Temperature is one of the main factors affecting the diffusion mass transfer process of CO_2_ and crude oil in fluids produced via CO_2_ flooding. The increase in temperature inhibits the diffusion mass transfer of CO_2_ molecules. The reason is that the increase in temperature makes the random motion of CO_2_ molecules more intense, thus increasing the distance between CO_2_ molecules, and oil and water molecules, and resulting in a decrease in the force between CO_2_ molecules, and oil and water molecules, which decreases the ability of CO_2_ molecules to undergo diffusion mass transfer.
(3) The gas–oil ratio changes greatly in the early and late stages of oilfield development, and its influence on the diffusion mass transfer of CO_2_ molecules cannot be ignored. An increase in the gas–oil ratio can enhance the E_CO2-oil_ and E_CO2-water_, making it easier for CO_2_ molecules to enter the oil and water phases, and thus promoting the diffusion mass transfer of CO_2_ molecules.
(4) When the water content changes, the distributions of the arrangements of oil and water molecules change greatly, which has a significant influence on the diffusion mass transfer process of CO_2_ molecules. The increase in the water content reduces E_CO2-oil_, thus decreasing the diffusion mass transfer of CO_2_ in the oil phase.

## Figures and Tables

**Table 1:** The diffusion coefficients of CO_2_ molecules at different temperatures.

Temperature (K)	283.15	293.15	303.15	313.15
Diffusion coefficient (10^−9^ m^2^/s)	5.67	6.33	7.17	7.83

**Table 2:** The diffusion coefficient of CO_2_ molecules at different gas–oil ratios.

GOR (m^3^/t)	287	574	1910
Diffusion coefficient (10^−9^ m^2^/s)	3.83	4.67	7.83

**Table 3:** The diffusion coefficient of CO_2_ molecules.

WC (%)	0	20	70
Diffusion coefficient (10^−9^ m^2^/s)	6.50	4.67	3.67

**Table 4:** Force field parameters.

	Force Field Model	Atom Type	ε/(kcal/mol)	σ/Å	q/e
CO_2_	EPM2-FLEX	C	0.0559	2.757	0.6512
		O	0.1599	3.033	−0.3256
C_n_H_2n+2_	OPLS	C(RCH_3_)	0.066	3.5	−0.180
		C(R_2_CH_2_)	0.066	3.5	−0.120
		H	0.03	2.5	0.06
H_2_O	TIP4P/2005	H	0	0	0.5879
		O	0.1852	3.1589	−1.1794

## Data Availability

Data are contained within the article.

## Nomenclature

symbol	parameter	unit
U	Total molecular potential energy function	/
U(r)	Van der Waals potential energy function	/
U(el)	Coulomb potential energy function	/
U(b)	Bond expansion potential energy function	/
U(θ)	Bond bending potential energy function	/
U(ϕ)	Bond torsion potential energy function	/
ε	The energy parameters	kcal/mol
$\varepsilon_{0}$	Reference energy parameters	kcal/mol
σ	The length parameters	Å
r	Distance between atomic pairs	Å
q	Charge of the atom	e
$r_{ij}$	Intermolecular distance of atoms i and j	Å
$k_{b}$	Elastic constant of bond stretching	Kcal/(mol·Å^2^)
$k_{\theta }$	Elastic constant of bond angle bending.	Kcal/(mol·rad^2^)
$\theta_{i}$	Bond angle	Å
ϕ	Proper dihedral angle	°
$V_{n}$	Force field parameters related to proper dihedral angle	/
$l_{i}^{0}$	Reference bond length	Å
$l_{i}$	Bond length	Å
$\theta_{i}^{0}$	Reference bond angle	Å
